# Fatal *Streptococcus iniae* Infection in a Juvenile Free-Ranging Short-Beaked Common Dolphin (*Delphinus delphis*)

**DOI:** 10.3390/ani11113123

**Published:** 2021-10-31

**Authors:** Rebecca Souter, Anne-Lise Chaber, Ken Lee, Aaron Machado, Jia Lam, Lucy Woolford

**Affiliations:** 1 School of Animal and Veterinary Sciences, The University of Adelaide, Roseworthy, SA 5371, Australia; rebecca.souter@student.adelaide.edu.au (R.S.); anne-lise.chaber@adelaide.edu.au (A.-L.C.); kennygeorgelee@gmail.com (K.L.); jiaernlam@hotmail.com (J.L.); 2 Australian Marine Wildlife Research and Rescue Organization, Torrens Island via Grand Trunk Way, Torrens Island, SA 5960, Australia; aaron@amwrro.org.au

**Keywords:** *Streptococcus iniae*, short-beaked common dolphin, zoonosis

## Abstract

**Simple Summary:**

*Streptococcus iniae* (*S. iniae*) is a significant aquatic bacterial pathogen that has caused devastating losses to wild and cultured fish populations worldwide and is an important zoonotic pathogen in humans. Previously reported in captive dolphins only, this case report describes *S. iniae* associated infection and mortality in a free-ranging short-beaked common dolphin (*Delphinus delphis*). Unreported previously in wild marine mammals, its detection highlights a need for further investigation into the epidemiology of *S. iniae* to better understand the risks for wild marine mammal populations and potential transmission cycles between marine mammals, humans and wild and intensive fish populations.

**Abstract:**

*Streptococcus iniae (S. iniae)* is a significant aquatic pathogen of farmed fish species, important zoonotic pathogen, and reported cause of disease in captive Amazon River dolphins (*Inia geoffrensis*) and a bottlenose dolphin (*Tursiops truncatus*). Here we report *S. iniae* as the cause of subcutaneous abscesses, sepsis and mortality in a juvenile free-ranging short-beaked common dolphin (*Delphinus delphis*) found deceased on a metropolitan Australian beach. Body surfaces were covered by multifocal, depressed, deep, irregular cutaneous ulcerations, which microscopically were characterised by ruptured subcutaneous abscesses with intralesional cocci. Routine microbiological investigations revealed a heavy growth of beta-haemolytic *Streptococcus* sp. identified as *Streptococcus iniae* in skin lesions as well as from heart blood, the latter supportive of sepsis. Tissues were negative for cetacean morbillivirus and no other disease processes were identified. *S. iniae* has not been reported in free-ranging marine mammals, nor in Australian delphinids, previously. More notably a pathogen of captive animals, this case report identifies *S. iniae* as a pathogen of wild dolphins also. In addition to expanding the host reservoir of a significant zoonotic pathogen, determining the source of infection as well as possible consequences for other marine mammals and wild and intensive fish stocks warrants further investigations.

## 1. Introduction

*Streptococcus iniae* (*S. iniae*) is a Gram-positive facultative anaerobe [1] considered a major pathogen in intensive aquaculture systems and free-ranging populations, causing disease in both fresh and saltwater fish [2,3,4]. In marine mammals, *S. iniae* was first isolated in Brazil in 1972 from a captive Amazon River dolphin (*Inia geoffrensis*) displaying numerous bilateral subcutaneous skin abscesses [1]. Subsequently, it has been identified in additional captive Amazon River dolphins in North America and as a cause of septicaemia and multisystemic inflammation in a captive adult bottlenose dolphin (*Tursiops truncatus*) in China [5,6,7]. *S. iniae* is also a significant zoonotic pathogen, with human disease first reported in 1991 [8].

Worldwide, approximately 27 species of fish have been reported with *S. iniae* infection [7,9], with sporadic outbreaks reported in tilapia (*Oreochromis niloticus*), seabass (*Dicentrarchus labrax*), Japanese flounder (*Paralichthys olivaceus*), rainbow trout (*Onchorynchus mykiss*) [2,3,10,11], and other species including various ornamental fish [7]. A major multi-species wild finfish mortality event was also attributed to *S. iniae* infection in Australia in 2016 [4]. Disease presentation is variable, with fish showing clinical signs including lethargy, anorexia, melanosis, and exophthalmia. Neurological signs can also occur with meningitis and meningoencephalitis following septicaemic progression of disease [2,9,12]. Mortality rates can be as high as 50% in these outbreaks, accounting for significant economic losses in aquaculture industries [10,13].

All previous reports of *S. iniae* infection in dolphins have been in captive settings; however, it is thought that disease may occur in free-ranging Amazon River dolphins [14]. Dolphins infected with *S. iniae* develop a dermatitis with multifocal subcutaneous skin abscessation, often referred to as “golf -ball” disease [1]. Suppurative enteritis, encephalitis, pancreatitis, and chronic pneumonia following septic progression of disease has also occurred in a Bottlenose dolphin (*T. truncatus*) [7]. Disease can resolve with treatment and improved husbandry practices, however, fatality is also recorded [1,6,7].

*S. iniae* is a zoonotic pathogen which causes cellulitis and bacteraemia in humans [7,12]. Septic progression of infection can result in endocarditis, meningitis, arthritis, pneumonia, toxic shock, and osteomyelitis [7,12,15]. While human infection may be opportunistic [9], it has been associated with recent handling and preparation of infected fresh fish and disease has occurred in various locations worldwide [7,8]. Current comprehension of *S. iniae* epidemiology highlights the potential increased risk of infection for individuals working in close proximity with marine species.

Here we report *S. iniae* associated disease and mortality in a free-ranging juvenile short-beaked common dolphin (*Delphinus delphis*). Previously unreported in wild marine mammals, detection of this agent and associated disease in a free-ranging individual highlights a need for further investigation into the epidemiology of *S. iniae* to better understand the risks for wild marine populations and potential transmission cycles between marine mammals, humans and intensive aquaculture operations.

## 2. Materials and Methods

A wild female short-beaked common dolphin was submitted to the University of Adelaide Veterinary Diagnostic Lab (UofA VDL) after being found dead on Aldinga Beach, South Australia, on the 22nd of August 2019 by a member of the public. Survey radiographs were taken prior to necropsy at the University of Adelaide Equine Health and Performance Centre in response to initial public concern over malicious injury. Necropsy occurred within 24 h of the cadaver being received and the specimen showed only mild autolysis. Necropsy was performed, photographed and recorded following routine protocol for small cetaceans (adapted from Pugliares et al., 2007) [16], with duplicate samples of major organs fixed in 10% formalin for histopathology or frozen pending further analysis. Swabs from skin lesions and heart blood were taken aseptically and submitted for aerobic and anaerobic culture at the UofA VDL, and MALDI-ToF identification was performed at the Australian Centre for Antimicrobial Resistance Ecology (ACARE), the University of Adelaide. Toxoplasma serological testing was performed at the University of Adelaide’s VDL using an indirect ELISA (ID Screen Toxoplasmosis Indirect Multi-species, Innovative Diagnostics, Grabels, France). Cetacean morbillivirus testing was performed on fresh lung at the Australian Centre for Disease Preparedness, Geelong, by TaqMan assay for both the 2000 Queensland and 2013 South Australian Isolates. Formalin fixed tissues were routinely processed for examination by light microscopy. After fixation for a minimum of 24 h, tissues were trimmed to 5-mm-thick sections and placed into labelled cassettes. Sections were dehydrated through graded ethanol concentrations, cleared with xylene, and embedded with paraffin wax and sectioned at 5 μm. Slides were stained with Harris’s hematoxylin and 1% eosin (HE), dehydrated, cleared, and mounted with a cover-slip using dibutyl phthalate pix xylene (DPX). Slides were examined and photographed using an Olympus BX43, DP25 camera and labSens software (Olympus corporation, Tokyo, Japan).

## 3. Results

### 3.1. Gross Necropsy Findings

The submitted juvenile dolphin weighed 13.6 kg with a straight snout to tail length of 116 cm, axillary girth of 54.5 cm, and dorsal, lateral and ventral blubber measurements 14 mm, 12 mm, and 14 mm, respectively. Survey radiographs revealed no radiodense projectile elements within the body walls or internal body cavities, and no evidence of fractures or other traumatic bone injuries. The dolphin was in thin body condition (BCS 2) [17] characterised by reduced epaxial muscle mass causing mild to moderate concavity ventrolateral to the dorsal fin as well as mild to moderate dorsal depression posterior to the blowhole.

**Figure 1:**
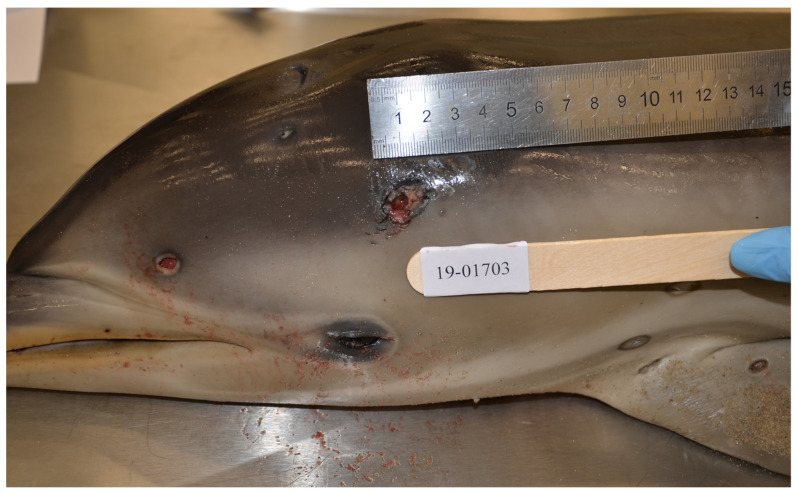
Female juvenile *D. delphis*. Multifocal non-raised deep irregular cutaneous ulcerations with necrotic centres which extend into the subcutis and blubber over the snout, lateral head, lateral body walls, and dorsal surface of the pectoral fins. The lesions vary in chronicity with variable depth and granulation tissue formation around wound edges, and range from 5 mm in diameter to 20 mm in diameter and up to 10 mm deep. Some lesions appear to be re-epithelialized/healed ulcers characterized by focal cutaneous depressions rimmed by a smooth edge of skin (dorsal and cranial to left pectoral fin).

**Figure 2:**
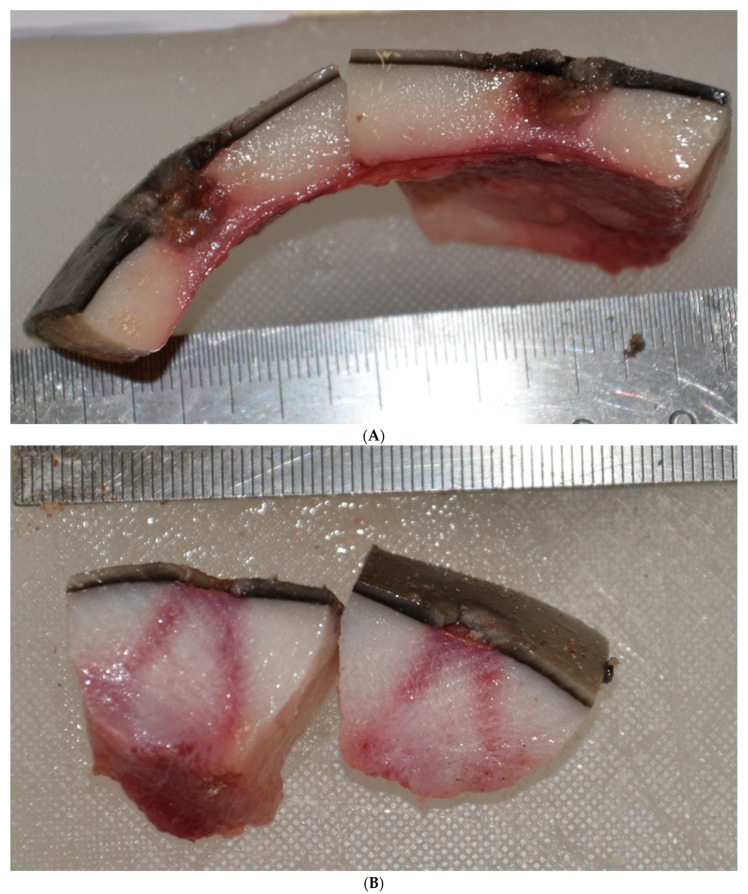
Female juvenile *D. delphis*. Bisected skin lesions showing extension of abscesses into the blubber layer, with rupture through the epidermis (**A**). Within the blubber layers beneath some lesion lines of erythema radiate deep to the ulcer (**B**), suggestive of congestion, inflammation, hemorrhage, and vasculitis with infarction.

Multifocal, deep, cavitating, cutaneous ulcerations 5–20 mm in diameter and up to 10 mm deep with necrotic centres extending into the blubber were noted over the skin of the snout, lateral head, lateral body walls, dorsal surface of the pectoral fins, dorsal tail and flukes (Figure 1 and Figure 2A). Linear wedge-shaped radiating lines of erythema were observed in the blubber below some lesions (Figure 2B), suggestive of vasculitis and dermal infarction. Lesions appeared at variable stages of chronicity, with healed ulcers characterised by smooth edged ulcers and re-epithelialized cutaneous depressions.

Stomach chambers were empty barring approximately 50 mL of yellow mucoid liquid in the pyloric stomach (C3). The rest of the gastrointestinal tract lacked any significant findings. The lungs were incompletely collapsed and there were depressed, mottled, red-brown, multifocal to coalescing areas of consolidation throughout the cranioventral and lateral aspects of the lung lobes.

### 3.2. Histopathological Findings

**Figure 3:**
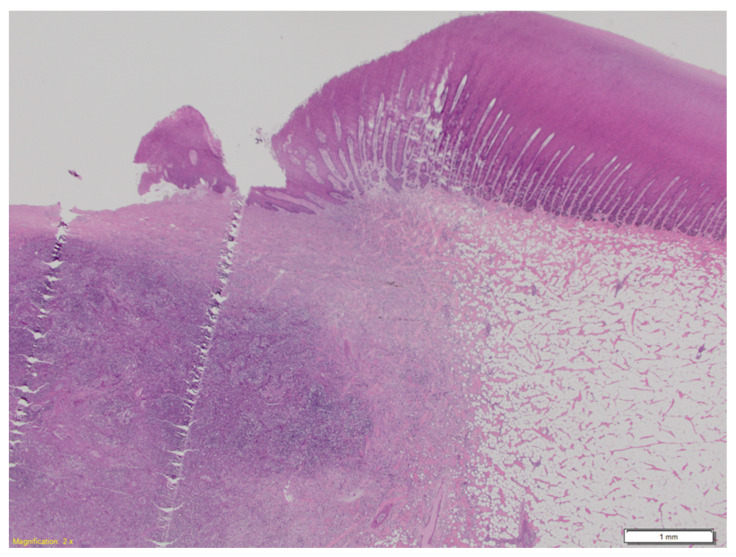
Female juvenile *D. delphis*, histopathological section of skin lesion from Figure 2A. A core of necrosuppurative inflammation surrounded by fibroplasia replaces the blubber layer, and erupts through the ulcerated epidermis. Hematoxylin and eosin, 2× magnification.

**Figure 4:**
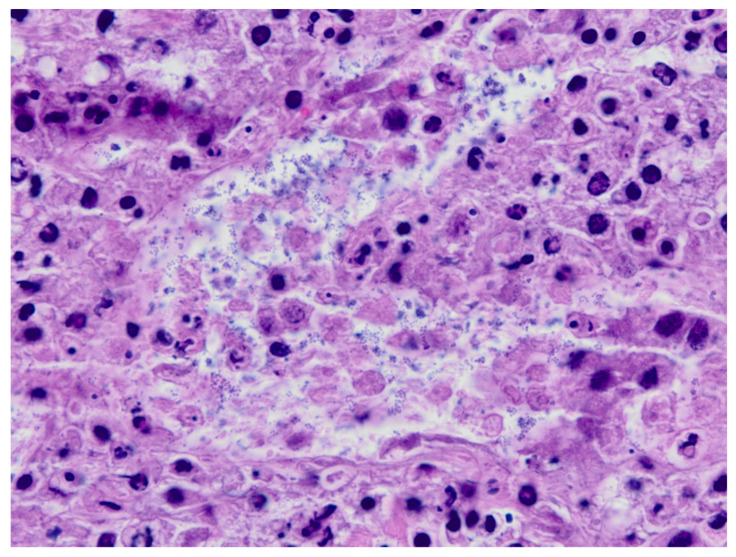
Female juvenile *D. delphis* Inset of skin lesion in Figure 3. Admixed with degenerate inflammatory cells are numerous bacterial cocci arranged in pairs, aggregates and rare chains. Hematoxylin and eosin, 100× magnification.

Microscopically, skin lesions were characterised by focal, irregular, extensive deep epidermal and dermal ulceration and loss with underlying subcutaneous foci of necrosuppurative inflammation (Figure 3) and intralesional cocci present as clusters and chains (Figure 4). In many sections of the adjacent skin, there was dermo-epidermal separation, and epidermal basilar degeneration and necrosis. Superficial dermal fibrin thrombosis and intravascular aggregates of cocci were also noted. Microscopically, grossly unaffected skin showed rare intravascular aggregates of cocci within superficial dermal vessels.

In the lung, alveolar interstitium was mildly to moderately hypercellular, characterized by increased circulating leukocytes within capillaries as well as mild to moderate interstitial infiltration of neutrophils, lesser macrophages and lymphocytes. In the adrenal gland, there were multifocal interstitial cortical haemorrhages and cocci were present in small cortical vessels, supportive of sepsis. Other findings included follicular hyperplasia and lymphocytolysis in Peyer’s patches of the colon, as well as mild lymphocytolysis in the spleen.

### 3.3. Microbiology and Viral Testing

Swabs from a skin lesion and the heart were submitted for bacterial culture; both swabs grew a heavy growth of beta-haemolytic *Streptococcus* sp. which were confirmed as *Streptococcus iniae* by MALDI-ToF analysis. A heavy growth of *Vibrio* sp. further characterised as *Vibrio pomeroyi* was also isolated from the skin lesions. Lung tissue was negative for both the 2010 Queensland and the 2013 South Australian CeMV isolates, and the dolphin was seronegative for Toxoplasma gondii infection

## 4. Discussion

Here we report the first detection of *Streptococcus iniae* and associated disease in a free-ranging Australian delphinid. This juvenile dolphin was found deceased in poor body condition with multifocal dermal and subcutaneous abscesses of varying chronicity, with gross and histopathological lesions and culture results supportive of death due to *S. iniae* associated sepsis. Skin lesions shared similarities with reported “golf-ball” disease in Amazon River dolphins.

Previous reports of *S. iniae* infection in dolphins have occurred in captivity [1,5,6,7], with multifocal subcutaneous abscesses the most common associated lesion [1,5,6]. Poor nutrition and unfavourable husbandry practices in captivity may contribute to septic progression of disease [6] and disease presentation can vary with bacteraemia due to possible immune suppression causing fatality without dermatological changes [7]. No testing for environmental pollutants or toxicants was performed in this case, therefore, cannot be excluded as a predisposing cause. Some *Streptococcal* spp. are considered commensal organisms with opportunistic pathogenic capacity, for example *Streptococcus phocae*, which alongside viral co-infection, has caused severe systemic disease in a common dolphin [18]. In cultured fish, *S. iniae* outbreaks have been associated with major stress events [19]. To understand the significance of skin bacterial pathogens as facultative (e.g., in immunocompromised hosts) or obligate, it is important to determine the normal skin commensal microbiome [18,20,21].

Given the subacute nature of the skin lesions, it is considered less likely that *S. iniae* associated illness [6] contributed to the muscle and adipose tissue atrophy (a chronic change occurring over a period of weeks); instead, it is more likely that other contributing stressors such as environmental disturbance or lack of feed led to wasting, immune suppression and subsequent increased susceptibility to infection [22]. As a juvenile, the impact of potential maternal illness or death must also be considered. Anthropogenic disturbance (e.g., boat and watercraft activity, habitat degradation, pollution) and climate events can instigate stress in a multitude of species. These stressors can result in immune collapse and subsequent increased opportunities for pathogenic bacterial colonisation [4,19,21]. CeMV was not detected in the lungs from this animal, however chronic CeMV infection and associated immunosuppression cannot be discounted on this basis alone and more extensive testing in future cases is indicated (serology, molecular analysis of a broader tissue range). Immunosuppression from alternative causes (such as toxicants and pollutants) may also have contributed to disease colonisation and progression.

*S. iniae* can spread hematogenously following initial infection [23]. *S. iniae* infection was elicited in captive barramundi (*Lates calcariferi*) through intraperitoneal inoculation, bath exposure and oral exposure [24]. In humans, injuries associated with fish preparation is the most common source of infection [9]. This may be extrapolated to cetaceans, and gives weight to the possibility of bacterial entry through consumption of reservoir fish or by waterborne sources entering percutaneous injuries [6,24]. In this case, the histopathological findings support septic progression of disease, with colonies of cocci consistent with *S. iniae* present within dermal vessels both in regions adjacent to and distance from skin abscesses, as well as internal organs such as the adrenal glands, associated with interstitial cortical haemorrhage in the latter. In the lung there was a mild to moderate mixed interstitial pneumonia, which was interpreted as supportive of systemic inflammation and sepsis. No viral inclusions or syncytia were observed to support viral disease. Final diagnosis was based on the pure growth of *S. iniae* from the heart blood swab and the dominant growth of the organism from the skin abscesses, and detection of morphologically consistent cocci microscopically within lesions.

The factors leading to *S. iniae* infection in this dolphin are not well understood, and climatic factors must be considered. Outbreaks of *S. iniae* infections in farmed fish have been linked to environmental fluctuations and rain fall events [25]. This dolphin was found deceased on a suburban beach near the end of winter (August, Southern hemisphere). Rainfall totals in August in the local region (Adelaide, South Australia) were near-average to below-average, however a record highest rainfall fell locally (Southern Vales) two weeks prior the dolphin being found; both daytime and night-time temperatures were cooler than average, with cold fronts crossing the city on a regular basis during the month (source Australian Bureau of Meteorology, www.bom.gov.au, accessed on 27 October 2021). Warming events were, therefore, unlikely, however the impact of rainfall and cold temperatures must be considered; in addition to the record rainfall event described above, a cold front brought hail to the region and light snow falls in the nearby Mount Lofty Ranges (rarely reported in this area) four days before the animal was found dead. Recently reported *S. iniae* associated mass fish mortalities in Western Australia were associated with extreme weather events in the region including a marked decrease in water temperatures, followed by an extended period of above-average coastal water temperatures [4]. More detailed studies on the impact of climatic conditions on South Australian marine mammal mortalities are presently underway.

The geographic distribution of *S. iniae* is poorly understood however, it has been reported in North America, Asia-Pacific, and the Middle East [9]. The bacterium has been isolated from fresh and saltwater cultured fish (e.g., *Lates calcarifer* and *Siganus* spp.) in northern Australia [24,26] and has caused mortality in multiple species of saltwater free-living finfish in north-west Australia [4]. *Delphinus spp.* are regularly reported in the Gulf St. Vincent, South Australia year-round [27]. They are social mammals and are often present in large groups [28], particularly juveniles [27]. It is unknown how *S. iniae* exposure occurred; however, the detection from an outer suburban beach will hopefully encourage future investigations into the health status of South Australian *D. delphis* populations and the presence of *S. iniae* within the ecosystem.

## 5. Conclusions

The diagnosis of *Streptococcus iniae* infection in a free-ranging short-beaked common dolphin within Australian waters is a novel finding and suggests the potential of *S. iniae* to spread and colonise Australian marine ecosystems. As well as being a significant zoonotic pathogen, *S. iniae* can have devastating impacts on cultured and wild fresh and saltwater fish. Everchanging environmental conditions influencing the immune status of wildlife can amplify the pathogenicity of bacteria. This highlights the need for further studies on *S. iniae* epidemiology to encourage the implementation of surveillance programs, disease awareness among communities, and improved management practices to prevent disease spread to humans and animals.
